# Reductions in development assistance for health funding threaten decades of progress in Africa

**DOI:** 10.1371/journal.pmed.1004695

**Published:** 2025-08-22

**Authors:** Peter MacPherson, Marriott Nliwasa, Augustine T. Choko

**Affiliations:** 1 School of Health & Wellbeing, University of Glasgow, Glasgow, United Kingdom; 2 Malawi-Liverpool-Wellcome Trust Clinical Research Programme, Blantyre, Malawi; 3 Kamuzu University of Health Sciences, Blantyre, Malawi; 4 Liverpool School of Tropical Medicine, Liverpool, United Kingdom

## Abstract

Countries across Africa face health crises driven by aid cuts, shifting demography, and infectious and environmental threats. Renewed public health strategies, smarter investment, and stronger surveillance can help, but reversing funding cuts is vital.

In the Malawian language Chichewa, the proverb *“Tsabola wakale sawawa”* is literally translated as *“Old pepper is not hot”* and can be interpreted as meaning that, as the world changes, new solutions are needed. For low-income countries like Malawi that are heavily dependent on donor aid, the world has indeed rapidly changed. Cuts of more than 83% have been made to the 2023 $42.4 billion USD budget of the US Agency for International Development (USAID), along with disruption to the President’s Emergency Plan for AIDS Relief (PEPFAR), as well as reductions in overseas aid development funding from countries like the United Kingdom [1]. These sudden cuts will have devastating consequences, with modeling indicating that USAID cuts will likely contribute to an additional 15.2 million AIDS deaths, 2.2 million tuberculosis deaths, 7.9 million child deaths, and 40–55 million unplanned pregnancies between 2025 and 2040 [2].

In a recent *PLOS Medicine* study [3], Molaro and colleagues set out to investigate how future declines in development assistance for health (DAH) to Malawi, and consequently health funding, will impact upon health projections for the country [3]. (It is worth noting that this research was undertaken before the recent substantial cuts to DAH funding and international aid). Specifically, they use a whole health systems model known as *Thanzi La Onse* to project the impact that different DAH funding scenarios would likely have on health outcomes in Malawi between 2019 and 2040. The modeling inputs are uniquely detailed, capturing evolving demography, risk factors for ill health, and incidence of a comprehensive set of infectious and non-communicable diseases, as well as health-seeking behavior, healthcare delivery, and intervention effectiveness. Importantly, the Ministry of Health of Malawi was closely involved in model development and checking, lending credibility to projections. The main finding is that overall increases in disability adjusted life years (DALY) should be anticipated for all likely DAH scenarios considered, ranging from a 7% to 16% increase compared to scenarios where health expenditure as a percentage of gross domestic product remains at current levels. In their analysis, the population benefits of increased health expenditure are clear, with reductions of ~10 million DALYs achieved for every percentage point increase in funding, but with diminishing returns above a 4% increase, due to capabilities constraints.

In contrast, projections across DAH growth scenarios for the three leading infectious diseases (tuberculosis, HIV/AIDS, and malaria) indicate that continued downwards trends in DALYs due to these diseases (particularly for TB and HIV/AIDS, but less so for malaria) can be sustained despite little or no projected growth beyond current health investment. This is likely because Malawi has already made substantial gains in achieving extremely high coverage of diagnosis and treatment of HIV [4], which has resulted in recent rapidly declining prevalence of TB [5]. This has been achieved through its pioneering “public health approach” to TB and HIV programming predicated on achieving scale-up through primary care, very limited treatment monitoring with expensive laboratory assays, and rigorous and responsive monitoring and evaluation programs [6].

However, complacency should be avoided; in the short term, the unprecedently large recent cuts to foreign aid budgets are likely to severely affect all programs, and especially those for TB, HIV, and malaria that are heavily aid-dependent, and will have far-reaching consequences for population health. The larger and longer the decline in international aid, the more expensive it will be to restore the health and prosperity of low- and middle-income countries. Moreover, interruptions to screening programs and diagnostic capabilities, as well as disruptions to treatment administration, monitoring, and evaluation capabilities may also result in re-emergence, as well as generation and transmission of drug-resistant disease that may go undetected for prolonged periods due to suboptimal surveillance systems.

The implications of these findings are stark, even before we consider the additional severe impact of the recent unprecedented cuts. To achieve sustained increases in life expectancy and avoid huge increases in ill health and disability in Malawi, funding dedicated to health must be increased. The authors note that while their analysis assumes “constant effectiveness” of medical interventions, technological innovation may lead to improvements in diagnosis and treatment, mitigating some of the more extreme projections. Although new diagnostics, treatments, and prevention have been developed for several major infectious diseases, deployment has been extremely inequitably distributed at both the global level as well as within regions and countries [7,8]. Innovation and implementation of new technologies for non-communicable diseases—the major contributors to projected increases in DALYs in this analysis—lags far behind. Action to bridging the “know-do gap” to improve accessibility and affordability of new technologies will be essential, and this will require concerted political and community advocacy [8].

So, what solutions could Malawi and other countries battling similar trends, and who have been suddenly and severely affected by recent health funding cuts, implement to mitigate against the worst effects, or indeed break this vicious cycle? At the national level, a renewal of the public health approach to prevention, health promotion, and healthcare delivery will be essential. Malawi has already shown that this can be done to tackle diseases like HIV and TB, and a renewed strategy centered around the major infectious and non-communicable diseases is required, focusing on maternal, infant, and child health, as well as the commercial, environmental, and social determinants of disease.

Efficient, high-quality surveillance, alongside monitoring and evaluation systems, can be effect-multipliers, increasing efficiency and supporting universal access to health. Focusing national infrastructure investment toward renewable energies, active transport, and digital technologies, while supporting sustainable agriculture and manufacturing, can yield large returns on investment, both in terms of future health, but also funding for health and other services. More broadly, cuts to DAH funding from countries in the Global North are antithetical to global commitments to equity, shared prosperity, and health security. If we are serious about preventing avoidable suffering, averting future pandemics, and achieving the Sustainable Development Goals, these cuts must not only be reversed, but accompanied by sustained, increased international assistance for health and development. Now is the time for renewed solidarity and investment—not retreat—to support countries like Malawi in building resilient, fair, and effective health systems.

## Abbreviations

**:**
DAH: development assistance for health
DALY: disability adjusted life years;
PEPFAR: President’s Emergency Plan for AIDS Relief
USAID: US Agency for International Development
